# CP-25, a Novel Anti-inflammatory and Immunomodulatory Drug, Inhibits the Functions of Activated Human B Cells through Regulating BAFF and TNF-alpha Signaling and Comparative Efficacy with Biological Agents

**DOI:** 10.3389/fphar.2017.00933

**Published:** 2017-12-22

**Authors:** Feng Zhang, Jin-Ling Shu, Ying Li, Yu-Jing Wu, Xian-Zheng Zhang, Le Han, Xiao-Yu Tang, Chen Wang, Qing-Tong Wang, Jing-Yu Chen, Yan Chang, Hua-Xun Wu, Ling-Ling Zhang, Wei Wei

**Affiliations:** Key Laboratory of Anti-inflammatory and Immune Medicine, Ministry of Education, Institute of Clinical Pharmacology, Anhui Medical University, Hefei, China

**Keywords:** BAFF, TNF-alpha, signaling pathway, CP-25, etanercept, rituximab

## Abstract

Paeoniflorin-6′-*O*-benzene sulfonate (code: CP-25) was the chemistry structural modifications of Paeoniflorin (Pae). CP-25 inhibited B cells proliferation stimulated by B cell activating factor belonging to the TNF family (BAFF) or Tumor necrosis factor alpha (TNF-alpha). CP-25, Rituximab and Etanercept reduced the percentage and numbers of CD19^+^ B cells, CD19^+^CD20^+^ B cells, CD19^+^CD27^+^ B cells and CD19^+^CD20^+^CD27^+^ B cells induced by BAFF or TNF-alpha. There was significant difference between CP-25 and Rituximab or CP-25 and Etanercept. CP-25 down-regulated the high expression of BAFFR, BCMA, and TACI stimulated by BAFF or TNF-alpha. The effects of Rituximab and Etanercept on BAFFR or BCMA were stronger than that of CP-25. CP-25, Rituximab and Etanercept down-regulated significantly the expression of TNFR1 and TNFR2 on B cell stimulated by BAFF or TNF-alpha. CP-25, Rituximab and Etanercept down-regulated the expression of MKK3, P-p38, P-p65, TRAF2, and p52 in B cells stimulated by BAFF and the expression of TRAF2 and P-p65 in B cells stimulated by TNF-alpha. These results suggest that CP-25 regulated moderately activated B cells function by regulating the classical and alternative NF-κB signaling pathway mediated by BAFF and TNF-alpha-TRAF2-NF-κB signaling pathway. This study suggests that CP-25 may be a promising anti-inflammatory immune and soft regulation drug.

## Introduction

B cells derive from bone marrow pluripotent stem cells and account for 15% of peripheral blood leukocytes (Tobon et al., 2013). B cells have lots of key roles, including presenting antigen, secreting pro-inflammatory cytokines and antibodies, and activating T cells (Namm et al., 2012; Bugatti et al., 2014). B cells activation and the loss of immune tolerance to self-antigens are involved in autoimmune diseases, such as rheumatoid arthritis (RA), systemic lupus erythematosus, and so on (Salazar-Camarena et al., 2016). The efficacy of B cell depletion therapy shows that B cells play a critical role in the pathogenesis of autoimmune diseases (Leandro, 2009).

In the peripheral blood, B cell subsets can be distinguished corresponding to different stages of differentiation, maturation and activation, which are characterized by the expression of different surface markers, such as CD19, CD20, and CD27 (Maecker et al., 2012; Leandro, 2013). CD19 is a special surface marker for B cells (Rossi, 2015). CD20 is expressed during B cell differentiation and lost during terminal differentiation to plasma cells. CD27 is a key marker of memory B cells and can promote memory B cells to differentiate into plasma cells (Han et al., 2016).

B cell activating factor belonging to the TNF family (BAFF), also known as B lymphocyte stimulator (BLyS), is an important cytokine of B cell survival, proliferation, and maturation (Wei et al., 2015; Zhang et al., 2016). BAFF is produced by monocytes, neutrophils, macrophages, activated T cells, dendritic cells, and so on (Yang et al., 2014). Over-expression of BAFF is involved in the pathogenesis of autoimmune diseases (Liu et al., 2011). BAFF can induce a series of intracellular response by binding to specific receptors. BAFF has three receptors: BAFF receptor (BAFFR), B cell maturation antigen (BCMA), and transmembrane activator and calcium modulator and cyclophilin ligand interactor (TACI) (Leandro and Cambridge, 2013). BAFFR can promote the maturation of immature B cells (Chong et al., 2014). BCMA mediates the survival and differentiation of plasma cells (Daridon et al., 2009). TACI can inhibit B cell expansion and facilitate the survival and differentiation of plasma cells (Zhang et al., 2015). BAFFR signal can activate the classical and alternative NF-κB signaling pathways, which are essential for B cell survival (Khan, 2009; Stohl, 2014). TACI and BCMA signal activates the classical NF-κB signaling pathway to counteract apoptosis (Liu and Davidson, 2011). The alternative NF-κB signaling pathway is mediated by the processing of NF-κB2p100 precursors to generate p52 (Coope et al., 2002). TNF receptor associated factors (TRAFs) function as adaptor molecules by associating with the intracellular domain of these proteins and subsequently mediating downstream signaling events. BAFF receptors can recruit one or more species of TRAF to their cytoplasmic domains (Vallabhapurapu et al., 2008). TRAF2 is a key intermediate in the classical and alternative NF-κB signaling pathways mediated by BAFF (Grech et al., 2004).

Tumor necrosis factor alpha (TNF-alpha), a pro-inflammatory cytokine, plays an important role in the development of B cells (Shaker et al., 2014). The serum level of TNF-alpha is elevated in patients with RA (Takeuchi et al., 2011). TNF-alpha is produced mainly by activated macrophages dendritic cells and T cells (Hundsberger et al., 2008). TNF-alpha exerts its biological functions via interaction with two receptors: TNFR1 and TNFR2. TNFR1 can induce apoptosis and TNFR2 promotes cell proliferation, migration (Pavkov et al., 2016). TNFR1 is widely expressed. TNFR1 mediates NF-κB activation and inflammatory response through the recruitment of TRAF2. NF-κB is activated by a sequence involving the phosphorylation and ubiquitination (Ma et al., 2016). The expression of TNFR2 is restricted to immune cells, TNFR2 lacks a death domain and mainly activates pro-survival signals by direct recruitment of TRAF2 and subsequently activates NF-κB (Gardam et al., 2008; Probert, 2015). However, it has not been clear whether the promotion of TNF-alpha on B cells maturation and differentiation through TNFR1/2-TRAF2-NF-κB pathway.

**FIGURE 1:**
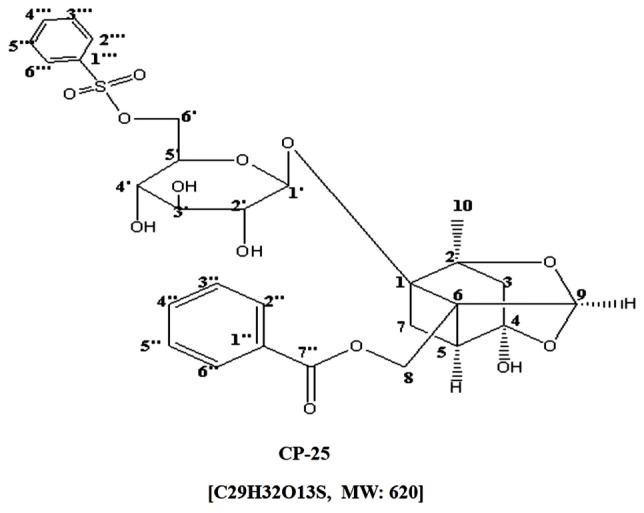
Chemical structures of CP-25.

Paeonia lactiflora Pall, a traditional Chinese medicine, has anti-inflammatory, antispasmodic and analgesic effects, and has been used for the treatment of RA for hundreds of years in China (Zhu et al., 2005). Total glucosides of paeony (TGP) was extracted from roots of Paeonia lactiflora Pall and approved for treating RA in 1998 in China. Paeoniflorin (Pae) is the effective natural active ingredient of TGP. TGP and Pae have anti-inflammatory and immunoregulatory effects, which have been extensively demonstrated in our laboratory (Chang et al., 2009, 2011; Wang et al., 2011; Jia et al., 2014). However, the low bioavailability of Pae limits its using in clinics. Therefore, in order to improve the bioavailability of Pae, a new active monomer paeoniflorin-6′-*O*-benzene sulfonate (code: CP-25; patent number in China: ZL201210030616.4) was got by the chemistry structural modifications of Pae in our laboratory (Yang et al., 2016). **Figure 1** shows the chemical construction of CP-25. The previous studies in our laboratory demonstrated that CP-25 significantly inhibited the progression of adjuvant arthritis rats by reducing inflammation, immunity, and bone damage primarily by modulating inflammatory mediators and immune responses, particularly Th17-IL-17 and CP-25 might repress the cell culture growth and cytokine secretion ability of fibroblast synovial cells (FLSs), and its inhibitory effects might be associated with its ability to inhibit the expression of BAFF-R in CD4^+^ T cells in a co-culture (Chang et al., 2016; Jia et al., 2016). At the same time, our team also found that CP-25 could inhibit the maturation of dendritic cells stimulated by TNF-alpha or Prostaglandin E2 (PGE2) through down-regulating the expression of MHC-II, CD40, CD80, CD83, and CD86 (Li et al., 2015), which suggested that CP-25 could regulate the function of immune cells. Therefore, some hypothesis are proposed. Whether CP-25 regulates the function of B cell, and whether CP-25 regulates B cell function through BAFF and TNF-alpha signaling pathways? It has been unclear. In this study, we aimed to investigate the effects of BAFF and TNF-alpha signaling pathway on the function of B cell and investigate the effects of CP-25 on activated B cells stimulated by BAFF and TNF-alpha *in vitro*. Meanwhile, we compared the differences of regulation of CP-25 on B cell functions with Etanercept or Rituximab.

## Materials and Methods

### Samples

Peripheral blood samples of healthy people from the First Affiliated Hospital Medical Center, Anhui Medical University were collected. The research followed the guidelines of the Declaration of Helsinki for humans. All healthy people gave written informed consent. All experiments were approved by the Ethics Review Committee for the Experimentation of the Institute of Clinical Pharmacology, Anhui Medical University (No. 20140186).

### Drugs and Reagents

CP-25 was supplied by the Chemistry Laboratory of Institute of Clinical Pharmacology of Anhui Medical University (Hefei, Anhui Province, China). Rituximab (Roche Pharma, Switzerland); Etanercept (Shanghai Guojian Pharmaceutical, Co., Ltd., China); BAFF (Pepro Tech, United States); anti-CD19-APC/PE/FITC, anti-CD20-FITC, anti-CD27-PE/APC, anti-BAFFR-APC, anti-BCMA-PE, and anti-TACI-PE (Biolegend, United States); anti-β-actin (ZSGB-BIO, China); Anti-TRAF2 (Santa Cruz, China); Anti-Phospho-NF-κBp65, anti-p100/52, and anti-Phospho-p38 MAPK (Cell Signaling Technology, United States); RPMI 1640 medium (Gibco, United States); fetal bovine serum (Zhejiang Tianhang Biotechnology, China).

### B Cell Magnetic Separation

B cells from peripheral blood mononuclear cells (PBMCs) were isolated using magnetic cell separation (MACS) by a positive selection. PBMCs (10^8^ cells) were incubated with PE-anti-CD19 for 20 min in 500 μL of MACS buffer, followed by incubation with anti-PE beads for additional 20 min. The stained cell suspension was applied onto LS column (Mitenyi Biotec, Germany). Labeled B cells were collected after washing degassed buffer for three times.

### B Cell Proliferation Was Detected by CCK-8 Kit

B cells (10^7^ cells/ml) were stimulated by BAFF (100 ng/ml) or TNF-alpha (100 ng/ml) for 2 h cultured in 96-well plates, and then were treated with CP-25 (10^-5^mol/l) or Rituximab (5 μg/ml) or Etanercept (10 μg/ml). After culture for 48 h, CCK-8 (10 μl) was added to each well. After incubation at 37°C in an atmosphere of 5% CO_2_ in air, The absorbance was measured by using an EJ301 ELISA Micro-well Reader at 450 nm.

### CD19^+^ B Cells, CD19^+^CD20^+^ B Cells, CD19^+^CD27^+^ B Cells, CD19^+^CD20^+^CD27^-^ B Cell, CD19^+^CD20^+^CD27^+^ B Cells, CD19^+^CD20^-^CD27^+^ B Cells Were Analyzed by Flow Cytometry

PBMCs (5 × 10^6^ cells/ml) from peripheral blood samples of healthy people were obtained by using human lymphocyte separation fluid and were stimulated by BAFF (100 ng/ml) or TNF-alpha (100 ng/ml) for 2 h, and then were treated with CP-25 (10^-5^mol/l) or Rituximab (5 μg/ml) or Etanercept (10 μg/ml). After culture for 48 h, PBMCs were stained with anti-human antibodies specific for anti-CD19-APC, anti-CD20-FITC, and anti-CD27-PE. After incubation for 30 min at room temperature in the dark, the cells were washed and immediately analyzed by flow cytometry.

### The Expression of BAFFR, BCMA, TACI on B Cells Was Analyzed by Flow Cytometry

Peripheral blood mononuclear cells were treated as the above methods and were stained with anti-human antibodies specific for CD19-PE/BAFFR-APC, CD19-APC/BCMA-PE, and TACI-PE. After incubation for 30 min at room temperature in the dark, the cells were washed and immediately analyzed by flow cytometry. BAFF receptors on B cells were defined as CD19 and BAFFR positive, CD19 and BCMA positive, CD19 and TACI positive.

### The Expression of TNFR1 and TNFR2 on B Cells Was Analyzed by Flow Cytometry

Peripheral blood mononuclear cells were treated as the above methods. The rabbit-derived TNFR1 antibody (1:100) or the mouse-derived TNFR2 antibody (1:100) was added into PBMCs at 37°C for 60 min, and then the FITC-labeled goat anti-rabbit secondary antibody (1: 50) or the FITC-labeled goat anti-mouse secondary antibody (1:50) or the PerCP/Cy5.5 goat anti-mouse secondary antibody (1:100) was added into PBMCs at 37°C for 30 min in the dark. Then the cells were washed and immediately analyzed by flow cytometry.

### The Expression of MKK3, P-p38, P-p65, TRAF2, p100/52 Was Analyzed by Western Blot

B cells (10^7^ cells/ml) were stimulated by BAFF (100 ng/ml) or TNF-alpha (100 ng/ml) for 2 h cultured at a density of 5 × 10^6^ cells/ml in 24-well plates and incubated at 37°C in 5% CO_2_ and then were treated with CP-25 (10^-5^ mol/l) or Rituximab (5 μg/ml) or Etanercept (10 μg/ml) for 24 h and then lysed in cell lysis buffer with 1 mM PMSF in the ice for 30 min. Debris was removed by centrifugation at 13622 *g* for 15 min at 4°C. The proteins from supernatant were separated by 10% sodium dodecyl sulfate polyacrylamide gel electrophoresis and transferred onto polyvinylidene fluoride membranes (PVDF Membrane, Millipore, United States). The membranes were blocked with the buffer (0.05% Tween 20-PBS with 5% non-fat milk) at 37°C for 2 h. Then the membranes were incubated with primary antibodies of rabbit monoclonal TRAF2 (1:500) or MKK3 (1:500) or P-p38 (1:500) or P-p65 (1:500) or P100/52 (1:500) and anti-β-actin (1:500) at 4°C overnight. The membranes were washed four times and then incubated with anti-rabbit or anti-mouse secondary antibodies conjugated with HRP (1:50000) for 2 h at 37°C. The detection was achieved by using the chemiluminescence reagent and exposed to film. Finally, the densities of the bands were quantified with a computerized densitometer (ImageJ Launcher, Broken Symmetry Software). Transfer efficiency and equivalent protein loading were verified by staining for β-actin.

### Statistical Analysis

In this study, there were five groups which were tested five times. One group was tested in triplicate. The percentage and numbers of B cell subsets were analyzed by flowjo software (United States). Relative protein quantification was done with Molecular Imaging software (United States). Data in figures are given as means ± standard deviation (SD). Analysis of variance (ANOVA) (SPSS Software Products, United States) was used to determine significant differences between groups. The criterion for significance was *P* < 0.05.

## Results

### BAFF and TNF-alpha Could Promote B Cells Proliferation, CP-25 Inhibited B Cells Proliferation

**FIGURE 2:**
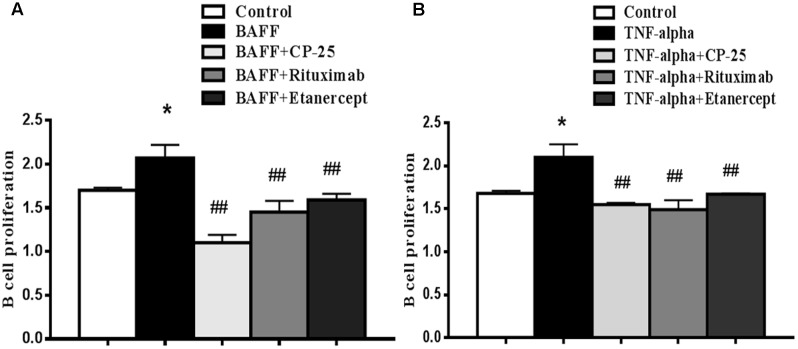
The effects of CP-25 on B cell proliferation stimulated by BAFF and TNF-alpha. B cells were stimulated with BAFF (100 ng/ml) **(A)** and TNF-alpha (100 ng/ml) **(B)** for 2 h, and then were treated with CP-25 (10^-5^ mol/l) or Rituximab (5 μg/ml) or Etanercept (10 μg/ml). After incubation for 48 h. B cell proliferation is assayed using the CCK-8 method. Bar graphs show the values of absorbance (450 nm) in different groups. Data are presented as mean ± SD (*n* = 5). ^∗^*P* < 0.05 versus control group. ^##^*P* < 0.01 versus BAFF/TNF-alpha group.

B cell proliferation was measured CCK-8 kit. Results showed that the proliferation of B cell was increased in BAFF group and TNF-alpha group, compared with control group. CP-25, Rituximab and Etanercept inhibited the increased B cells proliferation stimulated by BAFF and TNF-alpha. There was no significant difference among the three drugs (**Figure 2**).

### The Effects of CP-25 on the Percentage and Numbers of B Cell Subsets Stimulated by BAFF and TNF-alpha

**FIGURE 3:**
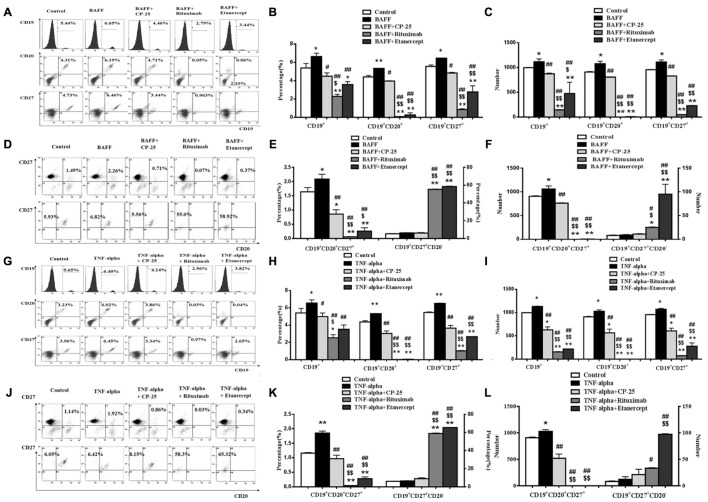
The effects of CP-25 on B cell subsets stimulated by BAFF and TNF-alpha. The expression of CD19^+^ B cells, CD19^+^CD20^+^ B cells, CD19^+^CD27^+^ B cells, CD19^+^CD20^+^CD27^-^ B cell, CD19^+^CD20^+^CD27^+^ B cells and CD19^+^CD20^-^CD27^+^ B cell is evaluated by flow cytometry. **(A**,**D,G,J)** The flow cytometry graphs are shown; **(B,E,H,K)** bar graphs show the percentage of B cell subsets in different groups; **(C**,**F**,**I**,**L)** bar graphs show the numbers of B cell subsets in different groups. The percentage and numbers is presented as mean ± SD (*n* = 5). ^∗^*P* < 0.05, ^∗∗^*P* < 0.01 versus control group, ^#^*P* < 0.05, ^##^*P* < 0.01 versus BAFF/TNF-alpha group, ^$^*P* < 0.05, ^$$^*P* < 0.01 versus CP-25 group.

The percentage and numbers of B cell subsets were analyzed by flow cytometry. Results showed that BAFF and TNF-alpha both significantly increase percentage and numbers of the CD19^+^ B cells, CD19^+^CD20^+^ B cells, CD19^+^CD27^+^ B cells, CD19^+^CD20^+^CD27^+^ B cells, compared with the control group. CP-25 reduced moderately the elevated percentage and numbers of CD19^+^ B cells, CD19^+^CD20^+^ B cells, CD19^+^CD27^+^ B cells, and CD19^+^CD20^+^CD27^+^ B cells induced by BAFF and TNF-alpha. Rituximab and Etanercept decreased significantly the percentage and numbers of CD19^+^ B cells, CD19^+^CD20^+^ B cells, CD19^+^CD27^+^ B cells and CD19^+^CD20^+^CD27^+^ B cells, which lead the above B cell subsets to be below the control level. Rituximab and Etanercept increased the percentage and numbers of CD19^+^CD20^-^CD27^+^ B cells. CP-25 had no effect on the percentage and numbers of CD19^+^CD20^-^CD27^+^ B cells. There was significant difference between CP-25 and Rituximab or CP-25 and Etanercept (**Figure 3**).

### The Expression of BAFFR, BCMA, and TACI on B Cells Was Up-Regulated by BAFF and TNF-alpha. CP-25 Down-Regulated BAFFR, BCMA, and TACI Expression

**FIGURE 4:**
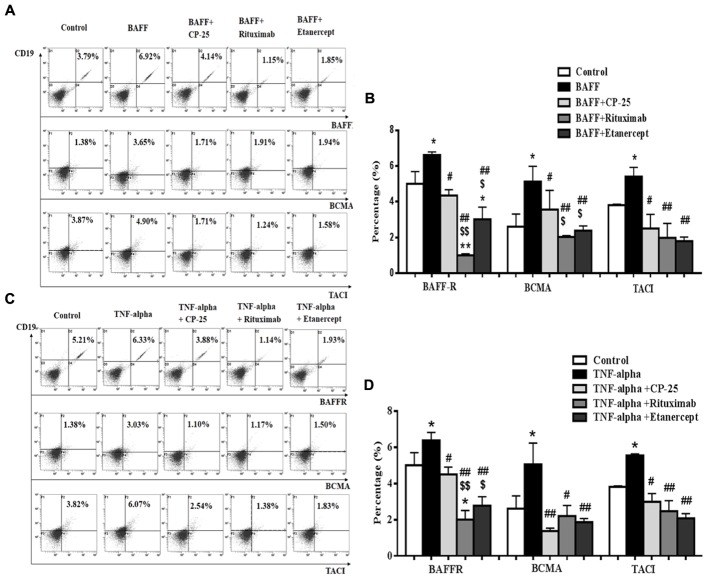
The effects of CP-25 on BAFF receptors (BAFFR, BCMA, TACI) on B cells stimulated by BAFF and TNF-alpha. The expression of BAFFR, BCMA, and TACI is analyzed by flow cytometry. **(A**,**C)** The flow cytometry graphs are shown; **(B**,**D)** bar graphs show the percentage of BAFFR, BCMA, and TACI in different groups. The percentage is presented as mean ± SD (*n* = 5). ^∗^*P* < 0.05, ^∗∗^*P* < 0.01 versus control, ^#^*P* < 0.05, ^##^*P* < 0.01 versus BAFF/TNF-alpha group, ^$^*P* < 0.05, ^$$^*P* < 0.01 versus CP-25 group.

BAFF receptors expression on B cells was analyzed by flow cytometry. Results showed that BAFF and TNF-alpha significantly up-regulated the expression of BAFFR, BCMA, and TACI, compared with the control group. CP-25, Rituximab and Etanercept could down-regulate significantly the high level of BAFFR, BCMA and TACI stimulated by BAFF and TNF-alpha. The inhibitory effects of Rituximab and Etanercept on BAFFR or BCMA were stronger than that of CP-25. Rituximab and Etanercept could induce the level of BAFFR to be below the control level. There was significant difference between CP-25 and Rituximab or CP-25 and Etanercept (**Figure 4**).

### BAFF and TNF-alpha Up-Regulated the Expression of TNFR1 and TNFR2 on B Cells, CP-25 Could Down-Regulate TNFR1 and TNFR2

**FIGURE 5:**
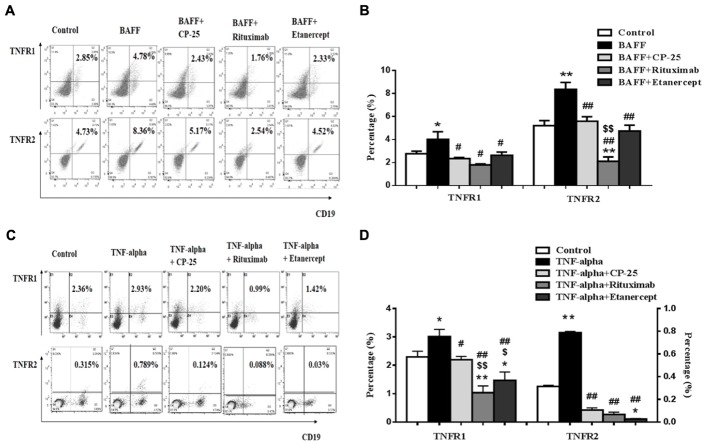
The effects of CP-25 on TNF-alpha receptors (TNFR1, TNFR2) on B cells stimulated by BAFF and TNF-alpha. **(A**,**C)** The flow cytometry graphs are shown; **(B**,**D)** bar graphs show the percentage of TNF-alpha receptors (TNFR1, TNFR2) in different groups, the percentage is presented as mean ± SD (*n* = 5). ^∗^*P* < 0.05, ^∗∗^*P* < 0.01 versus control, ^#^*P* < 0.05, ^##^*P* < 0.01 versus BAFF/TNF-alpha group, ^$^*P* < 0.05, ^$$^*P* < 0.01 versus CP-25 group.

TNFR1 and TNFR2 expression on B cells was analyzed by flow cytometry. Results showed that the expression of TNFR1 and TNFR2 was up-regulated in BAFF and TNF-alpha group, compared with control group. CP-25, Rituximab and Etanercept down-regulated the expression of TNFR1/2 on B cells stimulated by BAFF and TNF-alpha (**Figure 5**).

### CP-25 Could Inhibit the Expression of MKK3, TRAF2, P-p38, P-p65, and p100/p52 in B Cells Stimulated by BAFF and TNF-alpha

**FIGURE 6:**
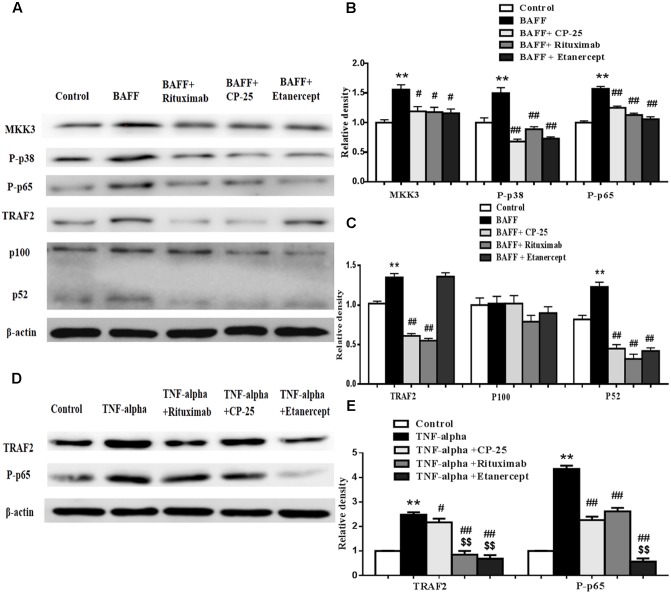
The effects of CP-25 on the expression of MKK3, P-p38, TRAF2, P-p65, and p100/52 in B cell stimulated by BAFF and TNF-alpha. The effects of CP-25 on the protein expression of MKK3, P-p38, TRAF2, P-p65, p100/52, TRAF2, and p100/52 **(A**–**C)** in B cell stimulated by BAFF are analyzed by western blot. The effects of CP-25 on the protein expression of TRAF2 and P-p65 **(D**,**E)** in B cell stimulated by TNF-alpha are analyzed by western blot. Data are presented as mean ± SD (*n* = 5). ^∗∗^*P* < 0.01 versus control group, ^#^*P* < 0.05, ^##^*P* < 0.01 versus BAFF/TNF-alpha group, ^$$^*P* < 0.01 versus CP-25 group.

The expression of MKK3, TRAF2, P-p38, P-p65, and p100/p52 in B cells was analyzed by western blot. Results showed that BAFF promoted the expression of MKK3, TRAF2, P-p38, P-p65 and p52 and TNF-alpha promoted the expression of TRAF2 and P-p65, compared with control group. CP-25, Etanercept and Rituximab could down-regulate significantly the elevated expression of MKK3, P-p38, P-p65, TRAF2, and p52 in B cells stimulated by BAFF. CP-25, Etanercept and Rituximab decreased significantly the expressions of TRAF2 and P-p65 in B cells stimulated by TNF-alpha (**Figure 6**).

## Discussion

Currently, the drugs for treatment of autoimmune diseases in clinic include non-steroidal anti-inflammatory drugs, steroidal anti-inflammatory drugs, disease-modifying antirheumatic drugs, biological agents and traditional Chinese medicines and natural medicines. These drugs generally play a therapeutic role by affecting the functions of immune cells involved in autoimmune diseases, including T lymphocytes, B lymphocytes, dendritic cells, and so on. In the pathogenesis of autoimmune diseases, the functions of immune cells are abnormally activated. It is an important effective mechanism that therapuetic drugs inhibit abnormal activation of the cells in the treatment of autoimmune diseases. However, adverse reactions of some drugs would occur under the conditions of inhibiting excessively immune cells function. Selective inhibitors targeting key molecules, including cyclooxygenase-2 inhibitors, CD20 monoclonal antibody, TNF-alpha inhibitors and so on, have serious adverse reactions in the treatment of autoimmune diseases (Fleischmann et al., 2012; Davies et al., 2015; Atzeni et al., 2016; Yun et al., 2016). Some active ingredients of traditional Chinese medicines and natural medicines exert soft regulation on the immune cell function. CP-25 is a new ester derivative of Pae, which is an effective natural active ingredient of TGP. This study found CP-25 might be a promising anti-inflammatory immune and soft regulation drug, which inhibited the functions of activated human B cells through regulating BAFF and TNF-alpha signaling.

B cells play an essential role by not only producing autoantibodies but also functioning as a source of cytokines and as antigen presenting cells that activate T cells in the pathogenesis of autoimmune diseases (Salinas et al., 2013). BAFF is known as a B cell survival and proliferation factor (de la Torre et al., 2010). In this study, BAFF promotes B cell proliferation. This finding is consistent with previous observation by Nguyen and Morris (2014). In their study, BAFF exerts a positive effect on maturation and proliferation of B cells (Nguyen and Morris, 2014). The number of B cells is also relative to BAFF level. Elevated BAFF level can lead to the expansion of the B cell pool, transitional and naive B cells are more dependent on BAFF (Kreuzaler et al., 2012). Our data showed that BAFF increased percentage and absolute numbers of total CD19^+^B cells, CD19^+^CD20^+^B cells, activated CD19^+^CD27^+^ B cells, memory CD19^+^CD20^+^CD27^+^ B cells. The biological role of BAFF is mediated by its receptors (Ng et al., 2004; Yu et al., 2012). The previous study found that BAFF increased TACI expression on CD19^+^ B cells *in vivo* (Nguyen and Morris, 2014). Our results showed that BAFF increased the expression of BAFFR, BCMA, and TACI on the B cells. Meanwhile, we also found that BAFF increased the expression of TNFR1 and TNFR2 on the B cells. BAFF-dependent survival and differentiation signals are dependent on TRAF2 (Rowland et al., 2010). TRAF2 plays an essential role in the regulation and homeostasis of immune cells (Etemadi et al., 2015). Our results showed that BAFF increased the expression of TRAF2 and P52, consisting with the previous study. In the study, BAFF could promote processing of the NF-κB2 precursor protein p100 to release p52 (Vallabhapurapu et al., 2008).

TNF-alpha is a potent inflammatory mediator and apoptosis inducer (Hildebrandt et al., 2015). TNF-alpha has an impact on B cell subsets and the subset of memory B cells was enlarged by TNF-alpha (Wang et al., 2013; Glaesener et al., 2014). The previous study in our lab indicated TNF-alpha promoted the up-regulation of TRAF2 and expression of TNFR1 on dendritic cells (Li et al., 2015). In our study, we found that TNF-alpha promoted B cells proliferation and TNF-alpha increased the percentage and numbers of CD19^+^ B cell, CD19^+^CD27^+^ B cells, CD19^+^CD20^+^CD27^+^ B cells. TNF-alpha increased the expression of TNFR1 and TNFR2. Meanwhile, we also found that TNF-alpha increased the expression of BAFFR, BCMA, and TACI on the B cells. TRAF2 serves as a positive signaling mediator in the TNFR pathways for the activation of downstream transcription factor (Cabal-Hierro and Lazo, 2012). In this study, B cell induced by TNF-alpha could express the high level of TRAF2 and P-p65.

The previous study in our lab indicated Pae inhibited the function B cells by PI3K/Akt/mTOR signaling pathway mediated by BAFF/BAFF-R (Li et al., 2012). In our study, CP-25 inhibited the percentage and numbers of B cell subsets, the BAFF receptors expression and TNFR1 and TNFR2 expression. CP-25 also reduced the high expression of P-p38, TRAF2, P-p65 and p52, which responded to B cells induced by BAFF. These results suggest that CP-25 regulates B cells function and inhibits the maturation of B cells induced by BAFF through the classical and alternative NF-κB signaling pathway mediated by BAFF. We also found that CP-25 was able to reduce the expressions of TRAF2 and P-p65 in B cells induced by TNF-alpha. These results suggest that CP-25 could regulate B cells function stimulated by TNF-alpha through TNFR1/2-TRAF2-p65 signaling pathway.

Here, we compared the effects of CP-25, Etanercept and Rituximab on B cell functions. Rituximab, a chimeric monoclonal antibody, depletes B cells by binding to the CD20 cell-surface antigen (Mease, 2008). Etanercept is a TNF-alpha antagonist. Rituximab and Etanercept are widely used to treat autoimmune diseases. In this study, Rituximab and Etanercept inhibited severely the function of B cells, and almost completely deleted B cell subsets of CD19^+^CD20^+^ B cells and CD19^+^CD27^+^ B cells and caused the percentage and numbers of CD19^+^ B cells, CD19^+^CD20^+^ B cells, CD19^+^CD27^+^ B cells and CD19^+^CD20^+^CD27^+^ B cells to be below the normal level. Severe inhibition effects of Rituximab and Etanercept on B cell function would lead to the dysfunction and apoptosis of B cells, which was one of mechanisms of adverse reactions for biological agents in the treatment of autoimmune diseases. CP-25 down-regulated moderately activated B cell subsets induced by BAFF and TNF-alpha, down-regulated BAFF and TNF-alpha receptor expression, inhibited BAFF/BAFF receptor mediated NF-κB signaling pathway and TNF-alpha-TNFR1/2-TRAF2-NF-κB signaling pathway to the normal level. Compared with Rituximab and Etanercept, the effect of CP-25 was modest, which suggests that CP-25 may be a promising anti-inflammatory immune and soft regulation drug.

## Conclusion

CP-25 regulated moderately activated B cells stimulated by BAFF and TNF-alpha to the normal level. CP-25 inhibited moderately activated B cell subsets induced by BAFF and TNF-alpha, down-regulated BAFF and TNF-alpha receptor expression, inhibited BAFF/BAFF receptor mediated NF-κB signaling pathway and TNF-alpha-TNFR1/2-TRAF2-NF-κB signaling pathway. The effects of Rituximab and Etanercept on the function of B cells were significantly stronger than that of CP-25. These results would be useful for clearing different pharmacological mechanisms of different drugs. This study further demonstrates the mechanisms of adverse reactions for biological agents in the treatment of autoimmune diseases, and also suggests that CP-25 may be a promising anti-inflammatory immune and soft regulation drug.

## Author Contributions

FZ and J-LS did the study, wrote this paper and analyzed data. YL, Y-JW, X-YT, CW, X-ZZ, LH, Q-TW, J-YC, YC, and H-XW contributed to the study. L-LZ and WW analyzed data and wrote the paper.

## Conflict of Interest Statement

The authors declare that the research was conducted in the absence of any commercial or financial relationships that could be construed as a potential conflict of interest.
